# Design of Miniaturized Double-Negative Material for Specific Absorption Rate Reduction in Human Head

**DOI:** 10.1371/journal.pone.0109947

**Published:** 2014-10-28

**Authors:** Mohammad Rashed Iqbal Faruque, Mohammad Tariqul Islam

**Affiliations:** Centre for Space Science (ANGKASA), Research Centre Building, Universiti Kebangsaan Malaysia, UKM, Bangi, Selangor D. E., Malaysia; University of California, Berkeley, United States of America

## Abstract

In this study, a double-negative triangular metamaterial (TMM) structure, which exhibits a resounding electric response at microwave frequency, was developed by etching two concentric triangular rings of conducting materials. A finite-difference time-domain method in conjunction with the lossy-Drude model was used in this study. Simulations were performed using the CST Microwave Studio. The specific absorption rate (SAR) reduction technique is discussed, and the effects of the position of attachment, the distance, and the size of the metamaterials on the SAR reduction are explored. The performance of the double-negative TMMs in cellular phones was also measured in the cheek and the tilted positions using the COMOSAR system. The TMMs achieved a 52.28% reduction for the 10 g SAR. These results provide a guideline to determine the triangular design of metamaterials with the maximum SAR reducing effect for a mobile phone.

## Introduction

Portable communication devices are widely used. Because the use of such mobile devices increases every year, an extensive study on the health risk from hazardous electromagnetic fields is currently in progress. The specific absorption rate (SAR) is the parameter used to evaluate power absorption in the human head. Radio frequency (RF) safety guidelines have been issued to prevent exposure to excessive electromagnetic fields in terms of the SAR [1]. The exposure of the human head to the near-field of a cellular mobile phone can be appraised by measuring the SAR in a human-head phantom or by calculating the exposure using a human-head numerical model [2].

Mobile phone radiation of the body's cells, brain or immune system has been suggested to elevate the threat of developing diseases, ranging from cancer to Alzheimer's disease. Laboratory tests on cockroaches have shown that radiation from mobile phones can have an adverse effect on overall health [3]–[5]. Note that research effort has also been dedicated to people suffering from headaches, fatigue and a loss of concentration after using their cellular mobile phones.

The study by Jensen and Rahmat-Samii considered a monopole, a side mounted Planar-Inverted F Antenna (PIFA), a top mounted curved inverted F-antenna and a back-mounted PIFA that utilized the FDTD method. They aimed to understand the effects of the tissue position and the corporal model on the antenna performance [6]. Gandhi et al. studied cellular telephones operating at 835 and 1900 MHz using a λ/4 and a 3λ/4 monopole antenna. They observed that the homogeneous model overestimated the SAR for a λ/4 antenna above a handset at 835 MHz with a radiation power of 600 mW [7].

Ref. [3] investigated the antenna efficiency, bandwidth, and SAR as a function of a mobile phone's armature-associated parameters, such as the length, thickness, width, and partition from the phantom. This statistical study established that the SAR increased while the radiation efficiency decreased when the resonant frequency of the armature equaled the resonant frequency of the antenna.

The effects of attaching conductive materials to mobile phones for SAR reduction were reported in ref. [8]–[10]. These studies showed that the position of the shielding material is an important factor for the effectiveness of SAR reduction. The spatial peak SAR needs to be reduced at the design stage of the material because the possibility of a spatial peak SAR exceeding the recommended exposure limit cannot be completely ruled out. The experiment described in [9]–[14] involved a perfect electric conductor (PEC) reflector positioned between a head model and folded loop antenna driver. The results of the experiment demonstrated that the radiation efficiency may be improved, and a decreased peak SAR value can be obtained. Such an antenna structure sacrifices the availability of signals received from all directions to the phone model. Ferrite sheet attachment reduced the SAR due to the suppression of surface currents on the front side of the phone model [10]. However, the relationship between the maximum SAR reducing effect and the parameters, such as the attaching location, size and material properties of the ferrite sheet, remains unknown. Furthermore, a bottom position is preferred to reduce the SAR. Moreover, SAR also depends on the type of handset or radiator used in a handset [15]–[18].

The SAR value due to a dipole antenna that is placed next to a plane phantom (flat phantom) was analyzed in ref. [19]. The authors demonstrated that the spatial peak of the SAR was directly related to the antenna's current distribution for frequencies above 300 MHz in the near field. Two relationships were discovered: (i) flanked by the SAR and the magnetic field and (ii) along with the SAR and the antenna's feed point current.

In [20], the authors analyzed the dependence of the exposure of the head on the antenna's radiation patterns using FDTD computations and compared the SAR using various head models. The authors demonstrated that the shaped-head model filled with a homogeneous liquid absorbed the most power and hence resulted in the highest SAR. The spherical model also exhibited a higher SAR than the anatomically correct model.

The main breakthrough of metamaterials is their ability to efficiently guide and control electromagnetic waves via the engineering of their material properties [21]–[24]. In recent years, significant research has been performed worldwide in order to study, develop and design metamaterials and their applications, particularly in electromagnetics. Metamaterials exhibit negative electrical permittivity and or negative permeability. When both the permittivity and permeability are negative, a metamaterial exhibits a negative refractive index, i.e. it is a left-handed material.

Different methods have been proposed over the last 20 years to reduce the SAR produced by emissions from handset antennas to levels below the current maximum exposure levels of the international standards, including auxiliary antenna elements, ferrite loading, EBG/AMC surfaces and low SAR handset antenna techniques. New results or ideas that promise useful devices for the future are being proposed each year. A number of detailed reviews and books have been published recently [25]. Among these results and ideas, the investigation of metamaterials is currently one of the most active topics in engineering and physics. Metamaterials are an emerging technology that has the potential to significantly change everyday life in the near future [26]–[28].

In particular, the problems to be solved in EM absorption reduction require an accurate representation of the mobile phone, anatomical representation of the head, alignment of the phone and the head, and an appropriate design of the metamaterials. Metamaterial techniques seem promising options to reduce the SAR in terms of low cost and ease-of implementation in mobile phones. This paper focuses on an antenna design that utilizes new metamaterial developments for SAR reduction.

In this paper, we emphasize artificial structures to design double negative TMMs that are attached to the PCB of mobile phones in order to reduce the SAR in the human head. This paper is structured as follows. Section 2 describes the numerical analysis of the handset in conjunction with the SAM phantom head. The modeling and analysis of the FDTD method coupled with the lossy-Drude model for SAR reduction are also discussed in Section 2. The impacts of the attachment of triangular split ring resonators (TSRRs) on SAR based on double negative TMMs are analyzed in Section 3. The design methodology of a TSRRs structure, the design simulation, and the fabrication of TMMs are explained in Section 4. Section 5 describes the experimental validation of the measurement results, and Section 6 concludes the paper.

## Model and Method

A simulation model that includes a handset with a PIFA-type antenna and the SAM phantom head provided by CST Microwave Studio (CST MWS) was used in this study. The numerical simulations were carried out using the finite integration technique (FIT) package in CST Microwave Studio. In these simulations, we added the losses that are typically associated with the resonant behavior of metamaterials, and the dielectric materials were considered perfect electric conductors. The relative permittivity and conductivity of the individual components were set to comply with industrial standards. In addition, the definitions in [13] were adopted for the material parameters involved in the SAM phantom head. To precisely characterize the performance over a broad frequency range, dispersive models for all dielectrics were used in the simulation [29]–[31].

The electrical properties of the materials used for the simulation are listed in Table 1. In the simulation model, a helical PIFA-type antenna was used, which is used for Global System for Mobile (GSM) 900 MHz applications. A high-quality geometrical approximation can be obtained for this structure from the meshing scheme of the FDTD method. The use of this meshing scheme in turn led to challenges in obtaining convergent results within a short simulation time.

**Table 1:** Electrical properties of the materials considered in the simulation.

Phone Materials	*ε_r_*	***σ*** (*S*/*m*)
Circuit Board	4.4	0.05
Housing Plastic	2.5	0.005
LCD Display	3.0	0.02
Rubber	2.5	0.005
SAM Phantom Head		
Shell	3.7	0.0016
Liquid @ 900 MHz	40	1.42

In this paper, the FDTD method with lossy-Drude polarization and magnetization models is used to simulate the DNG medium. Therefore, the permittivity and permeability are described as follows in the frequency domain:
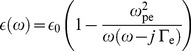
(1)

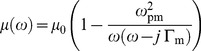
(2)where *ω_p_* and Γ denote the corresponding plasma and damping frequencies, respectively. Here, the Drude model is better suited for the FDTD simulations for both the permeability and permittivity functions. This approach provides a much wider bandwidth over which the negative values of the permittivity and permeability can be achieved. This option was only selected for numerical convenience and does not alter any conclusions derived from these simulations. Either case shows negative. However, choosing the Drude model for the FDTD simulation also allows significantly shorter simulation times, especially for low-loss media. In other words, the FDTD simulation will take longer to reach a steady state in the Lorentz model counterpart because the resonance region where the permittivity and permeability acquire their negative values would be very narrow in said model. With this method, we can treat the metamaterials as homogeneous materials with frequency-dispersive material parameters.

**Figure 1:**
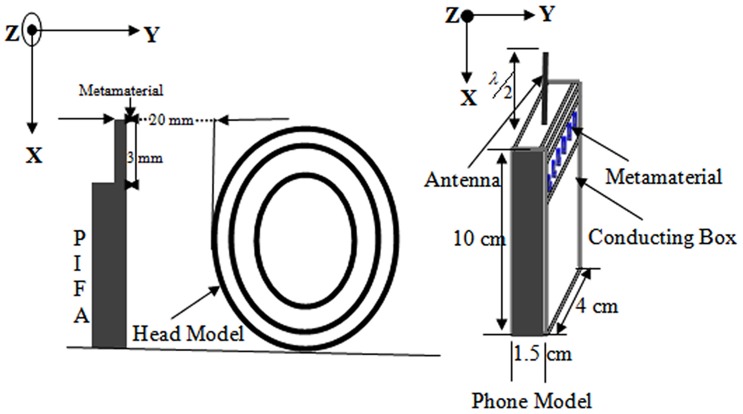
The head and antenna model for SAR calculation.

The handset featuring an antenna at 900 MHz considered in this study is shown in Fig. 1. This handset was modeled as a quarter-wavelength PIFA antenna positioned onto a rectangular conducting box that was 10 cm tall, 4 cm wide, and 1.5 cm thick. The PIFA antenna was placed on the top surface of the conducting box. A non-uniform meshing scheme was adopted to enable the major computational power to be dedicated to the regions along the inhomogeneous boundaries for rapid and flawless analysis. The minimum and maximum mesh sizes were 0.3 mm and 1.0 mm, respectively. The SAM head model used in this research consisted of approximately 2,097,152 cubical cells of 1 mm resolution. A time-step of 0.1 nanoseconds was used in the simulation, and the simulation duration was approximately eight sinusoidal cycles to ensure steady state conditions. The second-order Mur absorbing boundaries acting on the electric fields were implemented to absorb the outgoing scattered waves. An antenna excitation was introduced by specifying a sinusoidal voltage diagonally at the one-cell gap between the helix and the top surface of the conducting box.

The effectiveness of the SAR reduction and the antenna performance in different arrangements, sizes, and material properties of the materials and the metamaterials determined from the simulations are presented below. The head models used in this study were based on a MRI-based head model from the website named “whole brain Atlas”. Six types of tissues, bone, brain, muscle, eyeball, fat, and skin, were used in this model [12]. Numerical simulations of the SAR value were performed using the FDTD method. The parameters for the FDTD computation were defined as follows. The simulation domain was 128×128×128 cells. The cell sizes were set to Δ*x* = Δ*y* = Δ*z* = 1 mm. The computational domain was terminated with 8 cells of a perfect matched layer (PML). A PIFA antenna was modeled using the thin-wire approximation.

## Impact of the TSRRs Attachment on the SAR

In this section, the designed TSRRs were placed between a human head of a phantom and the antenna. This arrangement reduced the SAR value. To study this reduction at the GSM 900 band, different positions, sizes, and metamaterials were also analyzed by using the FDTD method to implement the detailed human head model.

The dispersive models for all dielectrics were used for the simulation to accurately analyze the TSRRs. The antenna was aligned to be parallel to the head axis. The distance between the antenna and the head was varied from 5 mm to 20 mm. Finally, a distance of 20 mm was chosen to compare the different metamaterials. The output power of the mobile phone model was set before the SAR was simulated. In this paper, the output power of the cellular phone was set to 600 mW at an operating frequency of 900 MHz. In the real world, the output power of the mobile phone will not exceed 250 mW for normal use, while the maximum output power can reach to 1 W or 2 W when the base station is far away from the mobile station (cellular phone). The SAR simulation results were compared with the results in [12], [30], and [31] for validation. When the phone model was placed 20 mm away from the human head model without a metamaterial, the simulated SAR 1 g peak value was 2.002 W/kg, and the SAR 10 g value was 1.293 W/kg. The level of SAR reduction was higher with metamaterial attachments. The results reported in [30] and [31] are 2.17 W/kg and 2.28 W/kg, respectively, for a SAR value of 1 g. These reported SAR levels are due to the mobile antenna position, i.e. the antenna structures were not properly aligned, and the antennae themselves also differed. These SAR values are significantly better than the result reported in [12], which was 2.43 W/kg for a SAR value of 1 g. This reported SAR value was achieved via different radiating powers and antenna designs.

The distance from the antenna feeding point to the edge of the metamaterial was A = 3 mm. The size of the metamaterial in the *x–z* plane was 62 mm×38 mm, and the thickness was 6 mm. The SAR value and the antenna performance with the metamaterial were studied. To calculate the antenna's radiated power, the source impedance (Z_S_) was assumed to be the complex conjugate of the free space radiation impedance (Z_S_ = 105.18+j81.97Ω). The source voltage (V_S_) was chosen such that the radiated power in free space is equal to 600 mW (V_S_ = 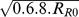). The source impedance and source voltage were held constant at the Z_S_ and V_S_ values when analyzing the effect of the metamaterials and the human head on the antenna performance.

The power radiated from the antenna was calculated by comparing the radiation impedance in this situation (Z_R_ = R_R_+jX_R_) with that using the following [14] equation:
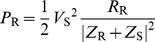
(3)The total power absorbed in the head was calculated using the following:
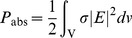
(4)


Different negative medium parameters were investigated to study the efficiency of the SAR reduction effectiveness. We positioned the negative permittivity media between the antenna and the human head to intercept the electromagnetic waves. Initially, the plasma frequencies of the media were set to *ω_pe_* = 9.239×10^9^ rad/s, which resulted in media with *μ* = 1 and *ε* = −3 at 900 MHz. Media with a larger negative permittivity, i.e., μ = 1, and *ε* = −5 or μ = 1 and *ε* = −7, were also analyzed. We set Γ*_e_* = 1.22×10^8^ rad/s, indicating that the media experience losses. The SAR 1 g peak level reduced to 1.0724 W/kg for media with *μ* = 1 and *ε* = −3, and metamaterials also affected the impedance. Compared to the control experiment without metamaterials, the radiated power was reduced by 14.55%, while the SAR was reduced by 46.44%. When traditional media are used, the SAR reduction effectiveness decreased compared to that of the metamaterials. In addition, the radiated power from the antenna was nearly unaffected when the metamaterials were used. Comparisons of the SAR reduction effectiveness for different positions and sizes of the metamaterials were performed.

The radiation pattern of the PIFA antenna combined with *μ* = 1 and *ε* = −3 metamaterials were analyzed to further examine whether the metamaterials affect the antenna performance. The radiation patterns were obtained from the near- and far-field transformation of the Kirchhoff surface integral representation (KSIR) [23]. All radiation patterns were normalized to the maximum gain attained without any added materials.

## Methods and Analysis of SAR for the proposed TSRRs

The SAR was reduced by placing the triangular metamaterials (TMMs) between the antenna and the head. Note that the TMMs were smaller than the operating wavelength and that the structures resonated due to their internal capacitance and inductance. The stop band can be tuned to the operational bands of cellular phone radiation. The TMMs were designed on a printed circuit board to allow them to be easily integrated into cellular phones. The overall dimensions of the TMMs were determined by a periodic arrangement of the sub-wavelength resonators.

### 1. Construction and Design of the TSRRs

**Figure 2:**
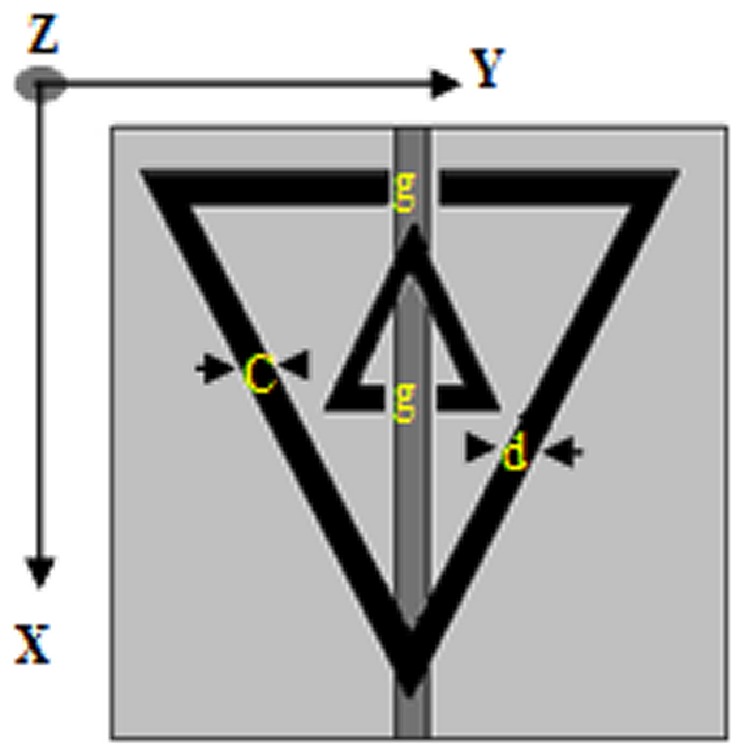
The structure of the TSRRs.

Using FDTD analysis, this research establishes that TMMs can reduce the 1 g SAR and 10 g SAR peak levels in the head. In this section, the TMMs are evaluated in a cellular phone 900 MHz band. Periodically arranged TSRRs can act as TMMs. The structure of TSRRs consists of two conductive material concentric triangular rings. Both triangular rings have a gap, and each ring is placed opposite to the gap of the other ring. The schematic of the structure of TSRRs used in this study is shown in Fig. 2.

To construct the TMMs for SAR reduction, the TSRRs were used as the resonator model, as shown in Fig. 2. The resonators operated in the 900 MHz band. The TSRRs consisted of two triangular rings, each with gaps on the opposite sides [9]. Note that the SRRs were introduced by Pendry et al. (1999) [22] and subsequently used by Smith et al. (2000) to synthesize the first left-handed artificial medium [21]. The metamaterials in this work were designed with periodic TSRRs arrangements to reduce the SAR value. By properly designing the TSRRs structure parameters, a negative effective medium parameter can be achieved for both the 900 MHz band.

The splits in the rings allow the SRR unit to resonate at wavelengths much larger than the diameter of the rings, i.e., the splits preclude the half-wavelength requirement for resonance incurred by closed rings. The purpose of the second split ring, which is split inside and whose split is oriented opposite to the first, is to generate a large capacitance in the small gap region between the rings, which considerably lowers the resonant frequency and concentrates the electric field. By combining the split ring resonators into a periodic medium to ensure a strong (magnetic) coupling between the resonators, unique properties emerge from the composite. In particular, because these resonators respond to the incident magnetic field, the medium can be viewed as having an effective permeability, μ*_eff_*(ω). However, we can use a physical approach and alter the dielectric function of the surrounding medium, which creates scattering properties that can distinguish whether the band gaps are due to either the μ*_eff_* (ω) or ε*_eff_* (ω) of the SRR being negative.

Combining the SRR medium that has a frequency band gap due to a negative permeability with a thin wire medium produces a left-handed material in the region where both μ*_eff_* (ω) or ε*_eff_* (ω) have negative values.

**Figure 3:**
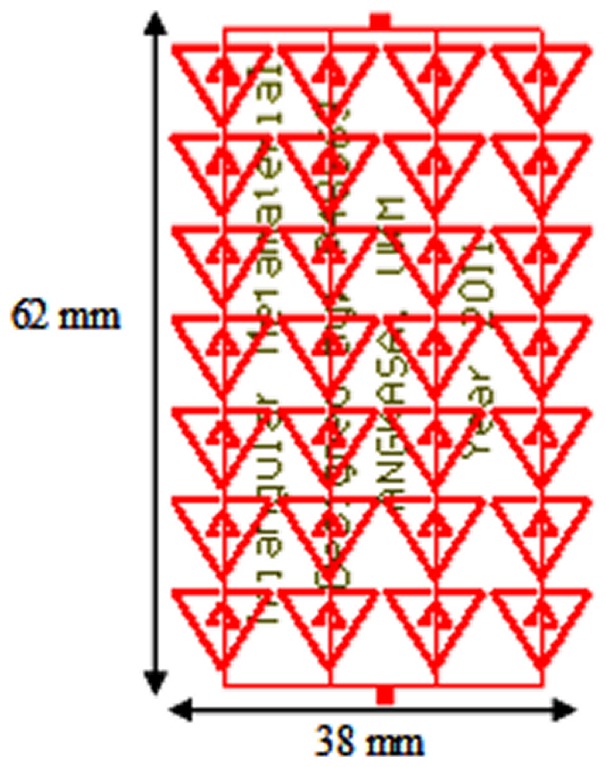
Arrays of TSRRs.

**Figure 4:**
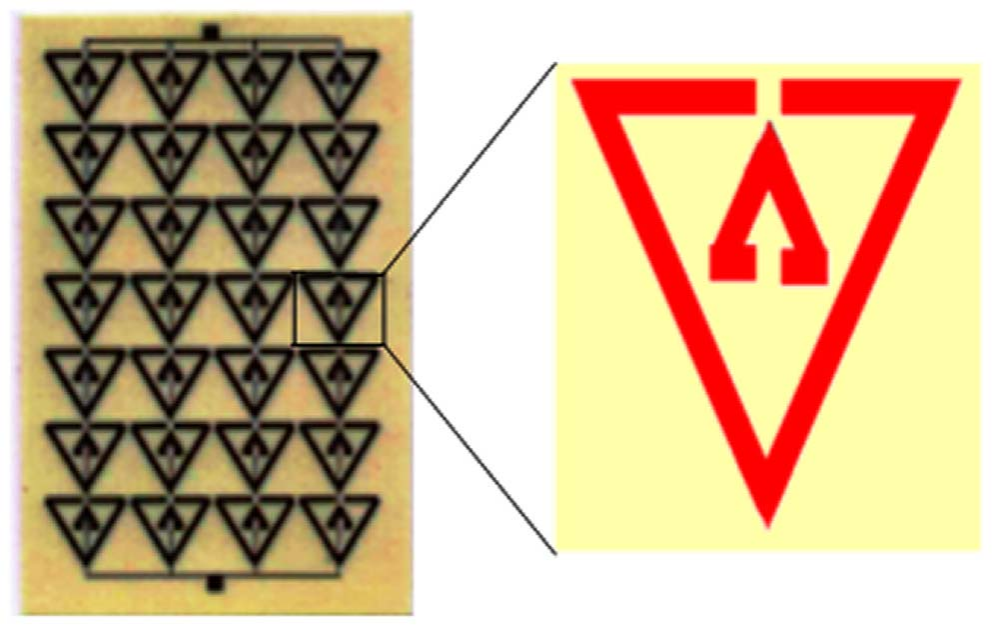
Fabricated TSRRs structure for the SAR measurement.

In Fig. 2, the resonator structures are defined by the following structure parameters: the triangular ring thickness, *c*, the triangular ring gap, *d*, the triangular ring size, *l*, and the split gap, *g*. Here, *c*_0_ is the speed of light in free space, and r is the radius of the inner ring. Fig. 3 presents the TSRRs arrays used for the SAR calculations in this work. These arrays were divided into four columns and seven rows. The dimensions of the TSRRs arrays considered in this work were 62 mm×38 mm. Fig. 4 shows the fabricated TSRRs used for the SAR measurement.

### 2. Results and Discussion

Numerical simulations that predict the transmission properties depend on the system's various structure parameters. Such complex simulations are typically performed by the FDTD method. Periodic boundary conditions can reduce the computational domain, while an absorbing boundary condition can be used to represent the propagation regions. The total-field/scatter-field formulation excites the plane wave. The regions inside the computational domain and those outside the TSRRs were set to be vacuums.

**Figure 5:**
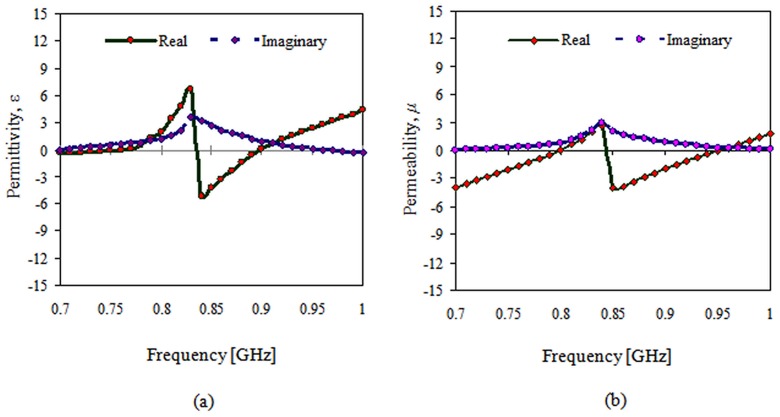
Materials measured at the resonant frequency are double negative: a) negative permittivity and b) negative permeability.

The permittivity and permeability are the two parameters that determine the metamaterial response to electromagnetic waves. At the resonant frequency of a double negative TMM, both the permeability and permittivity are negative, as shown in Fig. 5, which results in a negative refractive index. The permittivity and permeability can be determined by measuring the complex EM wave reflection and transmission coefficients of a material sample.

The figure shows that a region in the stop band exists where the permittivity and permeability is negative. Polarizing the magnetic field along the split ring axes, *_H_*_∥_, will generate a magnetic field that might either oppose or enhance the incident field. A bulky capacitance in the region among the rings will be generated, and the electric field will be strongly concerted. Strong field coupling was evident among TSRRs, and the permeability medium was negative at the stop band. In contrast, the magnetic field was parallel to the TSRRs plane for *H* ^⊥^.

Here, the author assumed small magnetic effects and a small, positive, and slowly varying permeability. In the *H* ^⊥^ condition, these structures can be viewed as sporadically arranging the metallic wires. The unremitting wires behave like a high-pass filter, which means that the permittivity can be negative underneath the plasma frequency.

The dissimilarity among the two stop bands in the *H*∥ and *H* ^⊥^ cases illustrates the difference among the magnetic and electric responses of the TSRRs. Theoretical investigations have shown that the *H*∥ band gap is due to negative permeability, and the *H* ^⊥^ band gap is due to negative permittivity. This study shows that both of the two incident polarizations can produce a stop band. In addition, a region in the stop band exists where the permittivity and permeability are negative. Polarizing the magnetic field alongside the split ring axes results in a magnetic field that may either resist or augment the incident field.

**Figure 6:**
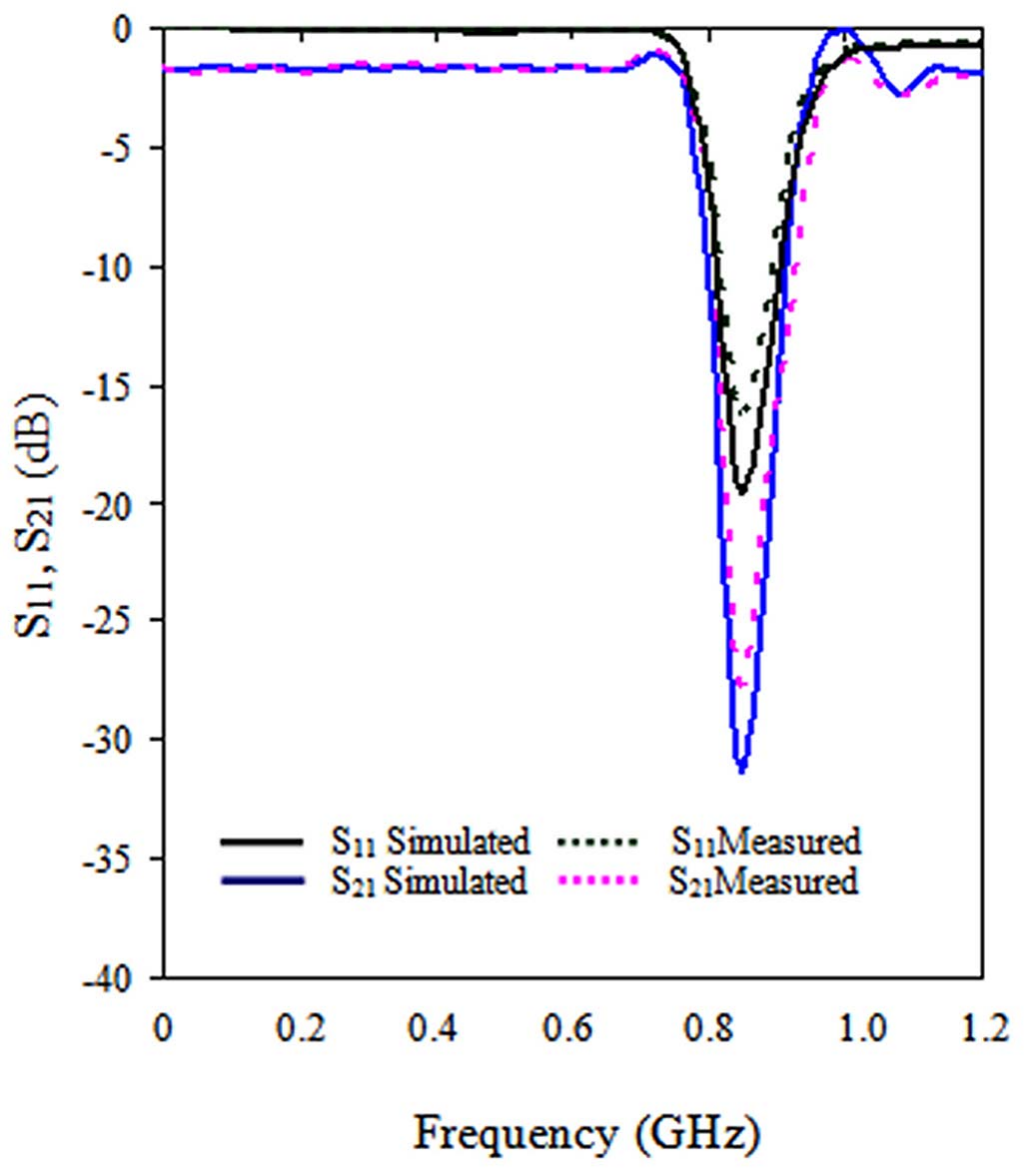
Modeled transmission spectra of the TSRRs plane in the *yz* plane.

To verify our FDTD simulation, the structure parameters of the TSRRs were set to those defined in [24]: *d* = *g* = *c* = 0.33 mm and *l* = 3 mm. The thickness and dielectric constant of the circuit board were 4.4 mm and 0.45, respectively. Twenty-eight unit elements were used in the propagation direction. Periodic boundary conditions were implemented normal to the direction of propagation. Fig. 6 illustrates the simulated and measured transmission spectra of the TSRRs studied. In [24], the measured results indicate that the SRRs exhibit a stop band extending from 8.1 to 9.5 MHz, whereas in this work, the simulation and the measured results indicate that the TSRRs exhibit a stop band extending from 8.3 to 9.4 MHz. This difference is due to the use of the square ring SRRs in [24] and the modified TSRRs with a novel TMMs shape used in this research.

The stop bands of the TSRRs were designed to be at 900 MHz and 1800 MHz. The periodicity along the *x*-, *y*-, and *z*-axes were *L_x_* = 62 mm, *L_y_* = 1.5 mm, and *L_z_* = 62 mm, respectively. To obtain a stop band at 1800 MHz, the TSRRs parameters were chosen to be *c* = 1.8 mm, *d* = 0.6 mm, *g* = 0.6 mm, and *r* = 12.7 mm. The periodicity along the *x*-, *y*-, and *z*-axes were *L_x_* = 50 mm, *L_y_* = 1.5 mm, and *L_z_* = 50 mm, respectively. The thickness and dielectric constant of the circuit boards for both the 900 MHz and 1800 MHz bands were 0.508 mm and 3.38, respectively. Once the geometric parameters were properly chosen, the TSRRs medium could exhibit a stop band at approximately 900 MHz and 1800 MHz.

## Experimental Validation

**Figure 7:**
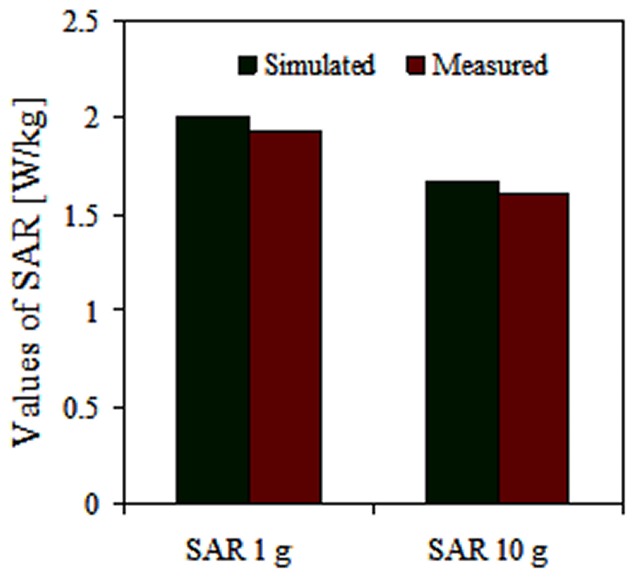
Comparison on SAR simulation and measurement results without the inclusion of TMMs.

The SAR measurement was performed using the COMOSAR measurement system. The system uses a robot to position the SAR probe inside the head phantom. The head phantom is filled with a liquid with dielectric properties selected based on IEEE standard 1528, which are *ε_r_* = 41.5 and *σ* = 0.97 S/m for 900 MHz and *ε_r_* = 40 and σ = 1.4 S/m for 1800 MHz. The measured and simulated SAR values without the inclusion of TMMs are shown in Fig. 7, and the distance between the head and phone model was 20 mm.

Fig. 7 indicates that the simulated SAR value is greater than 3.29% for the SAR value of 1 g and 3.82% for the SAR 10 g value because the distance between the head and phone model was not correctly calibrated for the measurement stage.

In addition, the distance between the source and the internal surface of the phantom position affects the SAR. For a 5 mm distance, a positioning uncertainty of ±0.5 mm would produce a SAR uncertainty of ±20%. Therefore, accurate device positioning is essential for accurate SAR measurements. In the measurement, the antennas with the TMMs are in contact with the SAM phantom head. During the measurement, the radiation power has been set to the maximum for the phone being tested (as required by the standard) of 33 dBm for GSM 900.

Moreover, peak SAR 1 g values of 2.002 W/kg and peak SAR 10 g values 1.293 W/kg were reached without the attachment of TMMS. When the TMMs were attached, the peak SAR 1 g value was 1.017 W/kg and the peak SAR 10 g value was 0.617 W/kg.

**Figure 8:**
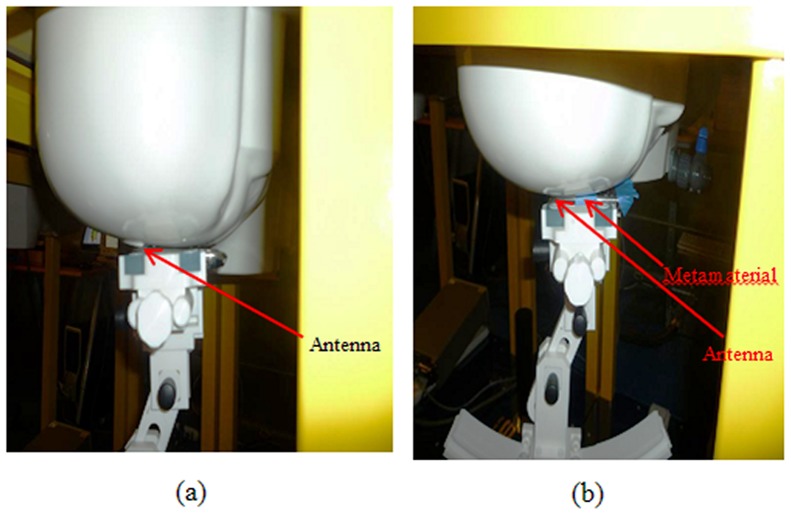
Measurement of the SAR values at the cheek position a) without a metamaterial and b) with a metamaterial.

**Figure 9:**
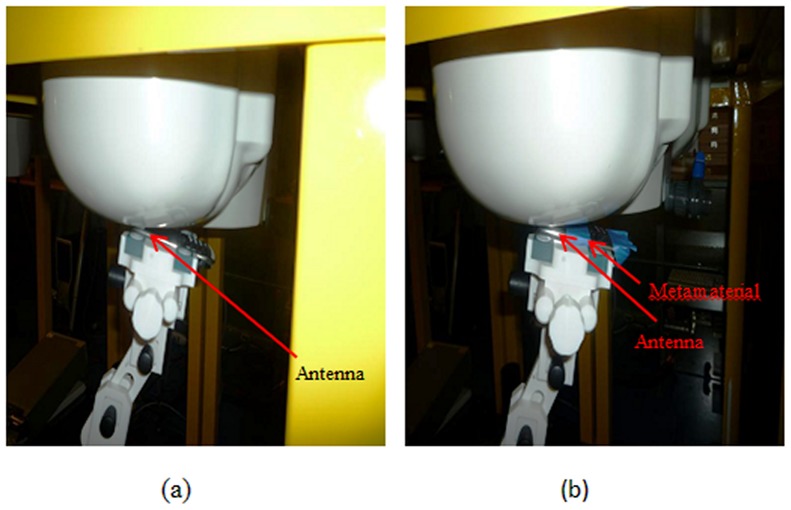
Measurement of the SAR values at the tilt position a) without a metamaterial and b) with a metamaterial.

Figs. 8 and 9 illustrate how to setup the apparatus for the cheek position and the tilted position measurements to determine the SAR value. Figs. 8 (a) and (b) show the cheek position of the measurement without metamaterials and with the attachment of TMMs. Fig. 9 (a) and Fig. 9 (b) show the 15° tilted position without metamaterials and with TMMS attachment.

**Figure 10:**
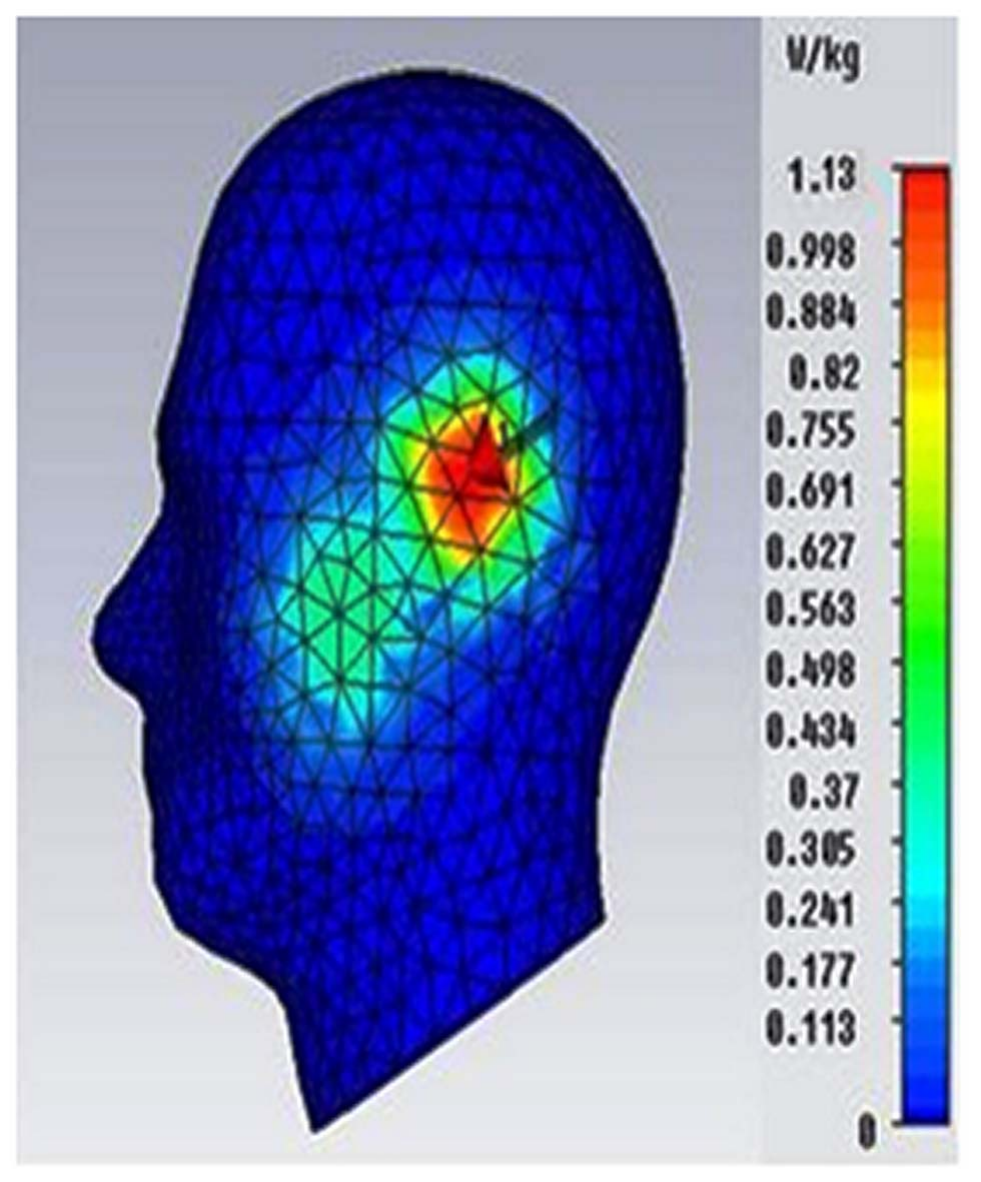
Simulated SAR value of the antenna with the TMMs attachment in the cheek position.

**Figure 11:**
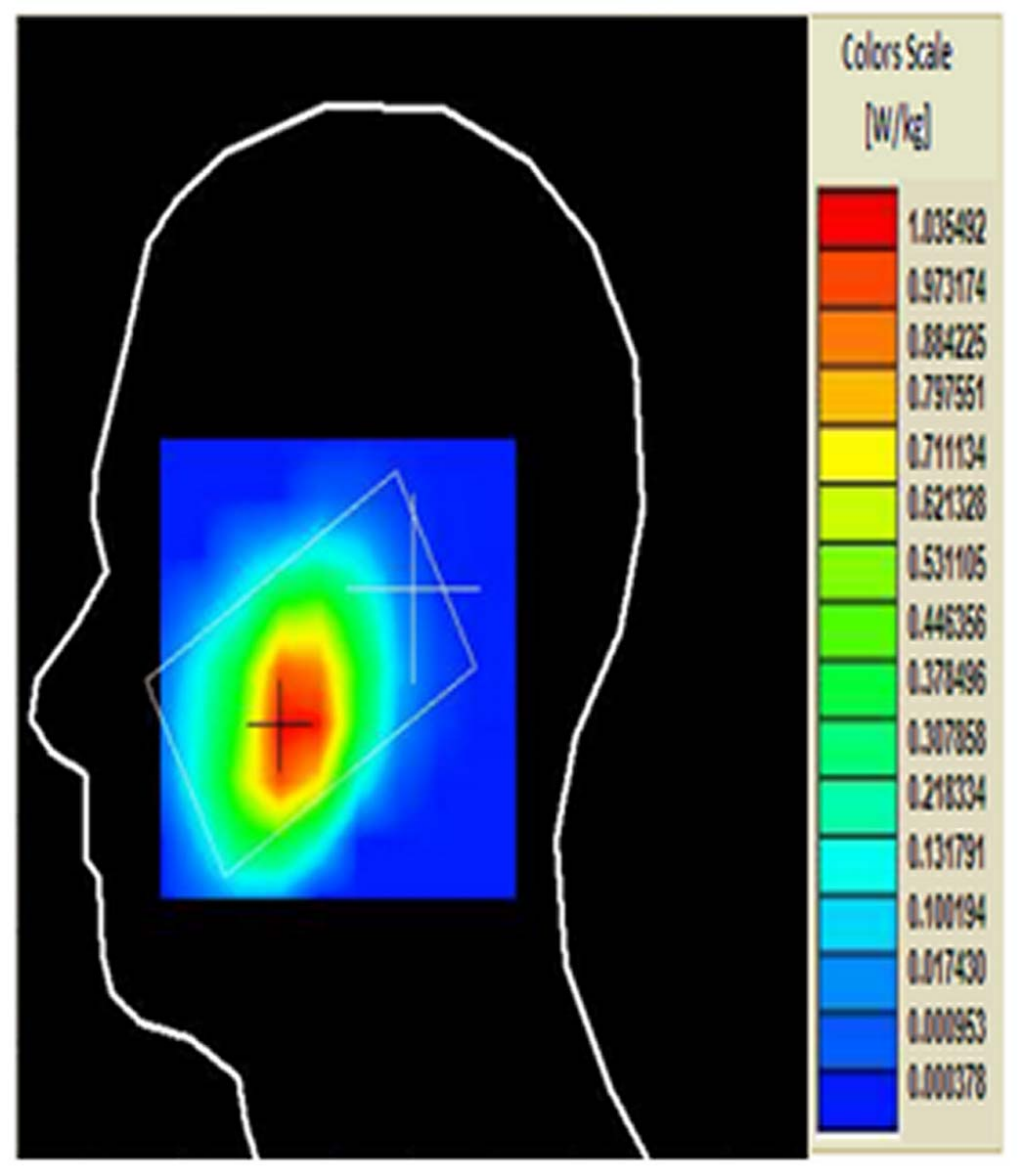
Measured SAR of the antenna with the TMMs attached in the cheek position.

Figs. 10 and 11 show the simulated and measured SAR values when the antenna with the TMMs attached was in the cheek position, which resulted in SAR 1 g values of 1.132 W/kg and 1.035 W/kg, respectively. The simulated and measured SAR differed by 9.70% for the SAR 1 g value. The higher SAR values measured for the cheek position than for the tilted position can be attributed to the influence of the ground plane on the distribution of the surface currents; consequently, the power deposited inside the head is higher at the GSM frequency band.

The simulated and measured SAR values were obtained using the tilted position for the TMMs with the antenna, which resulted in simulated and measured SAR 1 g values of 1.0963 W/kg and 1.017 W/kg, respectively, using the antenna with TMMs. The simulated and measured SAR values differed by 7.23% for the SAR 1 g value. Regarding the difference in the absolute values of peak SAR, the phone model casing for the simulation differed from the case used for the measurement. In addition, Scotch tape was used to attach the TMMs and the antenna during the measurement stages. The simulated and measured results also differ because the parameters for the measurement system change with the water evaporation and temperature. Furthermore, the measurement system contains several parameters (e.g. source, network emulator, probe, and electronic evaluation procedures) that affect the SAR calculation but are not included in the simulation.

**Figure 12:**
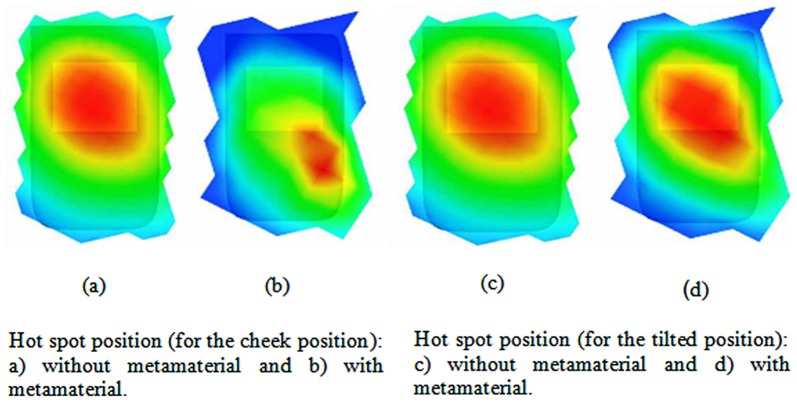
SAR comparisons at the different positions between an antenna without TSRRs and an antenna with TSRR_S_.

The SARs of the hot-spot positions without and with the attachment of the TMMs metamaterial for different positions are shown in Fig. 12 (a), (b), (c) and (d). The hot spots can be observed in the brain, especially in the region close to the eyeballs and at the muscle. Generally, hot spots are likely to occur at tissue interfaces with high dielectric contrast (i.e. fat and muscle tissue).

## Limitations of the Study, Research Questions and Future Work

The differences in the EM interactions between an antenna and the head caused by the double negative metamaterials have been discussed in this study. The developed TMMs parameters, such as the length, width, gaps and ring size, have been examined, and the frequency range of the negative *ε* and *μ* was analyzed. In addition, the simulated and experimentally measured SAR values were presented for the design of metamaterials.

A negative permittivity medium can also be constructed by periodically arranging the metallic thin wires. However, we found that when thin wires operated at 900 MHz are too large for practical application. Because the SRR structures resonate due to internal capacitance and inductance, they are smaller than the wavelength of radiation. Moreover, this study failed to reduce the SAR value for antennae that operate at 1800 MHz.

Some of the main issues that we intend to focus in this study include the following:

1. Is the existing material sufficient to reduce the SAR in the human body?
2. Are the proposed SRRs capable of effectively reducing the SAR?
3. Will the use of designed SRRs shrink the existing printed circuit board devices in mobile phones?

The field of metamaterials is relatively new, which translates into a very large scope for the further development of several factors presented in this study.

1. The development of metamaterials for wideband applications.
2. The use of PCB techniques to design the negative permittivity medium so that it can be directly implemented into portable devices.
3. The experimental realization of far-field sub-diffraction imaging at microwave wavelengths and the development of algorithms for complex sub wavelength structures.

## Conclusion

The electromagnetic energy absorption reduction between an antenna and the human head has been discussed. The proposed double-negative metamaterials in the phone model achieved a 10 g SAR value of 0.617 W/kg and a 1 g SAR value of 1.0175 W/kg. Based on the FDTD method with the lossy-Drude model, both the 1 g SAR and 10 g SAR peak values of the head can be abridged by placing metamaterials between the antenna and the human head. The use of all dielectric metamaterials tuned to the exact *ε* and *μ* values required that the electromagnetic energy be diverted from the cellular phone user's head, which are promising options to improve the SAR reductions. The achieved results provide constructive information for the design of communication equipment that complies with the safety requirements.
